# Retraction Note: Cisplatin-induced azoospermia and testicular damage ameliorated by adipose-derived mesenchymal stem cells

**DOI:** 10.1186/s40659-026-00697-8

**Published:** 2026-05-13

**Authors:** Hamdy Y. Ismail, Nora A. Shaker, Shaymaa Hussein, Adel Tohamy, Mohamed Fathi, Hamdy Rizk, Y. R. Wally

**Affiliations:** 1 Department of Anatomy and Embryology, Faculty of Veterinary Medicine, Cairo University, Giza, Egypt; 2 Department of Cytology and Histology, Faculty of Veterinary Medicine, Cairo University, Giza, Egypt; 3 Department of Toxicology & Forensic Medicine, Faculty of Veterinary Medicine, Cairo University, Giza, Egypt; 4 Department of Theriogenology, Faculty of Veterinary Medicine, Cairo University, Giza, Egypt

**Retraction Note to: Biological Research (2023) 56:2**

10.1186/s40659-022-00410-5

The Editor-in-Chief has retracted this article. Two panels in figure 5 appear to overlap at different magnifications, even though they are supposed to represent the same animal at different time points. Different measurements are labelled in each panel despite their overlap. The authors did not provide a sufficient response to concerns. The Editor has lost confidence in the data and conclusions of this article.

Author Hamdy Youssef disagrees with this retraction. All other authors did not respond to correspondence from the Publisher about this retraction.

